# ESHO 2–85. Hyperthermia as an adjuvant to radiation therapy in the treatment of advanced neck nodes: A randomized multicenter study by the European Society for Hyperthermic Oncology

**DOI:** 10.2340/1651-226X.2024.41035

**Published:** 2024-12-12

**Authors:** Jens Overgaard, Olav Dahl, Giorgio Arcangeli

**Affiliations:** a Department of Experimental Clinical Oncology, Aarhus University Hospital, Aarhus, Denmark; b Department of Oncology and Medical Physics, Haukeland University Hospital, Bergen, Norway; c Department of Clinical Science, University of Bergen, Bergen, Norway; d Regina Elena National Cancer Institute, Rome, Italy

**Keywords:** Squamous cell carcinoma of the head and neck, neck nodes, hyperthermia, radiotherapy, randomized multicenter trial, quality assurance

## Abstract

**Background and purpose:**

European Society for Hyperthermic Oncology (ESHO) 2–85 is a multicenter randomized trial investigating hyperthermia (HT) as an adjuvant to radiotherapy (RT) in treatment of locally advanced neck nodes. The trial never fulfilled recruitment and was stopped prematurely, and has not previously been published.

**Patients and methods:**

Between January 1987 and February 1993, 64 evaluable neck nodes in 54 patients were included. Tumors were stratified according to institution and nodal size and randomly assigned to receive RT alone (2 Gy/fx, 5 fx/wk) to a total dose of 60–70 Gy, including boost, or the same RT followed once weekly by HT (aimed for 43°C for 60 min). The primary endpoint was persistent complete response (local control).

**Results and interpretation:**

Sixty-four tumors in 54 patients were evaluable, with a median observation of 17 months. Thirty-four tumors were randomized to RT alone and 30 to RT+HT. Compliance with RT was good. HT was associated with moderate to severe pain and discomfort in 38% of the treatments. In 57% of the heated patients at least one treatment achieved the target temperature. HT did not significantly increase radiation morbidity. The complete response rate was 53% in the RT versus 80% in the RT+HT group, and 3-year persistent local control rate was 32% for RT alone versus 53% for RT+HT; HR: 0.48 [0.23–0.98].

The ESHO 2–85 study demonstrated that addition of a weekly HT treatment to RT of advanced neck nodes significantly enhanced the persistent tumor control. The results substantiate the potential clinical benefit of hyperthermic oncology.

## Introduction

Throughout the late 1970s and early 1980s an intense effort was made to explore the clinical potential of using moderate hyperthermia (HT) to enhance the effect of radiotherapy (RT). This happened through numerous smaller uncontrolled studies, which strongly suggested that adjuvant HT may increase the probability of controlling tumors by RT [1–5]. At the same time, the biological rationale and practical scenario for combining HT with radiation was developed [5].

The clinical application of heat was (and still is) hampered by the difficulties to provide a homogeneous heating to a given target. Despite these shortcomings, abundant clinical experience of combined heat and radiation was gathered, and overall pointed towards a significant benefit of combining heat with radiation relative to radiation alone [5–7]. Furthermore, the early clinical studies also indicated that the improvement in local tumor control could be obtained without increased normal tissue morbidity if either a relatively long interval between the two modalities was allowed for, or if the tumors were heated (semi)selectively. These explorative studies were mainly performed on superficially located tumor sites where heating was possible, such as advanced neck nodes, recurrent or advanced breast cancer, and malignant melanoma, although pelvic tumors were also included. This initial clinical experience indicated that in advanced neck nodes from squamous cell carcinoma of the head and neck (HNSCC) an improvement in local control of could be obtained with a thermal enhancement ratio in the order 1.5 [5]. The HNSCC associated neck nodes, has been one of the most exploited superficial tumor sites, and the early studies have demonstrated a significant thermal enhancement, the success of which may be influenced by the quality of the heating and the nodal volume [8–11].

Based on this uncontrolled, but promising experience, the European Society for Hyperthermic Oncology (ESHO) initiated several prospective phase III clinical trials [12, 13], of which all but one have been finally reported [14–18]. The current article describes the remaining study targeting advanced neck nodes, which is presented in full for the first time. The trial was designed to include 120 tumors, but due to poor recruitment the study was prematurely ceased after 3 years. Since the launch of our study, several randomized studies involving HNSCC and neck nodes have made a similar evaluation and confirmed our hypothesis [19–25].

The ESHO protocol 2–85 (enclosed as supplementary material) is a multicenter randomized clinical trial with the objective to assess the efficacy of local HT given as an adjuvant to primary RT of advanced neck nodes from HNSCC, to evaluate the nodal tumor response and control probability, assess early and late tolerance in normal tissues, and evaluate the feasibility of available heating techniques. A weekly interval between the heat sessions was applied to avoid the problems of thermotolerance [26]. Such a schedule will primarily take advantage of the hyperthermic cytotoxicity rather than the more powerful hyperthermic radiosensitization, which demands true simultaneous (and homogeneous) application to be fully utilized [5, 7].

Since the study was performed more than 30 years ago, several of the participating departments and hospitals no longer exist and most investigators are not professionally active anymore. Thus, it is impossible to obtain missing/additional information and the current report may suffer from such shortcomings. However, we feel an ethical commitment to publish the results, in order to comply with the legal requirement to report the outcome of clinical trial results [27, 28], and in respect for the willingness of the patients to participate in medical research. Despite the incompleteness and long history of the trial, the data still strengthens the scientific evidence of moderate HT as an enhancer of ionizing radiation and point to the need to further exploit the potential of hyperthermic oncology [1].

## Patients and methods

### Patients and pretreatment evaluation

This multicenter randomized trial included patients with locally advanced neck nodes who were candidates for primary RT provided they fulfilled the following criteria: The primary tumors should be squamous cell carcinomas originating in the head and neck area (HPV/p16 status was not determined). The nodes should be clinically classified as N1–N3 (UICC classification 1982). All lesions should be considered feasible for heating with the available equipment. The patients should have a life-expectancy > 6 months, accept participation in the study and frequent follow-up should be possible. The tumor should be measured in at least 2 and if possible 3, diameters together with the depth from the skin surface. CT-scanning, or ultrasound should be performed to give an objective measurement of tumor size and depth. Patients with multiple (bilateral) nodes may be included. If so, one of the nodes should be assigned to HT. Otherwise, the patients were evaluated according to normal department policy.

### Trial design

Patients fulfilling the given criteria were stratified according to institution and tumor size (*N* ≤ 3 cm vs *N* > 3 cm) and randomized to one of the following schedules:

Conventional radiotherapy alone (RT).

or

Conventional radiotherapy as shown, followed once weekly with HT (RT+HT).

The study was designed as a proof of principle and feasibility trial evaluating the outcome in the neck nodes specifically. The heating technique did only permitted treatment to the N-site nodes and not to the primary T-site tumor. Irrespective of the randomization arm the T-site was only treated with RT and consequently the influence on more patient relevant endpoints (NED [no evidence of disease], overall survival, etc.) could only be evaluated indirectly, through the extent that the outcome in the neck-node may have on the overall disease. Furthermore, multiple neck-nodes in some patients were randomized to different treatment.

### Treatment

RT was given with high-voltage photons according to the departments’ general policy. Radiation dosimetry was as specified in ICRU report 29 [29]. The given tumor dose to the nodal and primary tumor areas should be 60 Gy. An additional boost up to 10 Gy through reduced portals may be given to the residual tumor. The primary tumor should receive at least the same dose as the nodes. Radiation should be delivered in daily fractions of 2 Gy, 5 fractions per week.

HT was applied with electromagnetic heating. There were no limitations to the equipment used except that it should be likely to provide a tumor temperature of 43.0°C. Active skin cooling was allowed. The heat treatment should be applied once weekly in the first 5 weeks and should be started within half an hour after irradiation. Each heat session should aim for a minimal tumor temperature 60 min at 43.0°C or equivalent. Efforts were made to avoid heating of normal tissue or to apply appropriate skin cooling. HT and thermometry (with multiple measurement points in both tumor and normal tissue) were performed and recorded in accordance with the ESHO quality assurance guidelines [30]. In addition to the actual temperatures and times recorded, these values were also calculated as cumulative equivalent minutes at 43°C (CEM43) [31]. If the tumor or disease in general did not successfully respond to treatment or otherwise required so, additional treatment could be given according to department policy.

### Follow-up and evaluation

Patients were planned to be seen with regularly intervals, as indicated in the protocol. Given the limitations in heat treatment, the primary study endpoint was persistent local control in the neck nodes (3-years rates of local control). Secondary endpoints were complete tumor response (CR), acute and late normal tissue damage, NED, disease-free and overall survival. Tumor response was defined according to WHO [32]. Early and late radiation damage was recorded as previously described [18]. In addition, the importance of treatment quality should be evaluated.

### Statistical and ethical considerations

The known dose-response relationship for both radiation alone and combined radiation and HT treatment of advanced neck nodes [5, 7] indicate that a treatment gain in the order of 30% improvement in local control was to be expected. The trial was, therefore, designed to be closed after 120 evaluable lesions had reached at least a 3-month follow-up. If the true frequency of any event was changed by 30%, (e.g. from 50% to 80%), the likelihood that a significant difference being observed (*p* < 0.05) would be more than 90%. The treatment effect was evaluated using ‘the intention to treat’ principle and evaluable lesions were included in the randomization group irrespectively of whether, or not, they had completed the planned treatment.

Local control, NED, and disease-free survival (DFS) were estimated by the Kaplan–Meier product-limit analysis using the Log-rank test for comparison. Outcomes were measured from date of randomization. Local control was defined as persistent disappearance of the involved neck nodes in the treated area. NED was defined as time to any first failure (loco or regional failure or distant metastasis). DFS was defined as time to the first failure (loco or regional failure or distant metastasis) or death from any cause. Events occurring after 3 years were censored at 3 years. The Cox proportional hazards model was used to assess hazard ratios reported with 95% CI.

The study was designed according to the requirements laid down by the Helsinki Declaration II [27]. Patients were informed about the aims, methods, anticipated benefits and potential hazards. The protocol was adapted to and approved by all relevant national or local ethical committees.

## Results

### Patients and tumors

**Figure 1:**
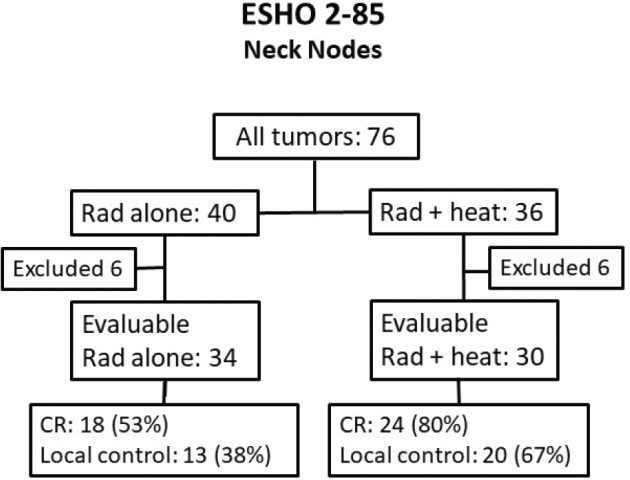
Consort flow diagram showing tumor and outcome as a function of randomization group.

Patients were recruited between January 1987 and February 1993. At the time of closure of the trial, a total of 76 tumors in 64 patients were included (12 patients had simultaneous bilateral tumors). Twelve tumors in 10 patients (6 in each randomization arm) were excluded either because the patients died before treatment (2 pts), refused any treatment and/or follow-up despite randomization (2 tumors), or no treatment and follow up data were reported. This left 64 evaluable tumors in 54 patients with a median age of 63 (range 38–83) years, and with a median observation time of 11 (range 1–55) months. The dominating primary T-site was pharynx, and 13 of the nodes occurred in previously treated patients, whereas 51 nodes were in de novo primary treated patients. Thirty-four tumors were randomized to RT alone and 30 to RT+HT. Size below 3 cm was 8 and 7 of 34 RT and 30 RT+HT treated tumors, respectively. Figure 1 and Table 1 provide an overview of the evaluable patients and tumor characteristics and show the two treatment groups to be comparable.

**Table 1:** Characteristics of 64 evaluable tumors in 54 patients*.

Parameter	RT	RT+HT
All tumors*	34	30
Nodal size		
Median vol (cm^3^)	17 (1–166)	16 (2–154)
≤ 3 cm	8	7
> 3cm	26	23
Primary treatment	27	24
Recurrent nodes	7	6
Primary T-site		
Oral cavity	4	9
Larynx	4	2
Pharynx	25	16
Other	1	3
Age (years)		
Median (range)*	62 (38–83)	64 (38–82)

RT: radiotherapy; HT: hyperthermia.

* 5 patients had simultaneous tumors, treated with RT and RT+HT, respectively.

### Tumor response

Fifty-five (90%) of the treated nodes obtained a complete or partial response, which in most patients was persistent. The combined heat and radiation treatment yielded a significantly higher complete response rate than tumors treated with radiation alone (Table 2).

**Table 2:** Initial tumor response in 64 evaluable tumors.

Endpoint	RT (%)	RT+HT (%)	All tumors (%)
No response (NR)	7 (21)	2 (7)	9 (14)
Partial response (PR)	9 (26)	4 (13)	13 (20)
Complete response (CR)*	18 (53)	24 (80)	42 (66)

RT: radiotherapy; HT: hyperthermia.

* *P* < 0·01, Fisher’s exact probability test.

**Figure 2:**
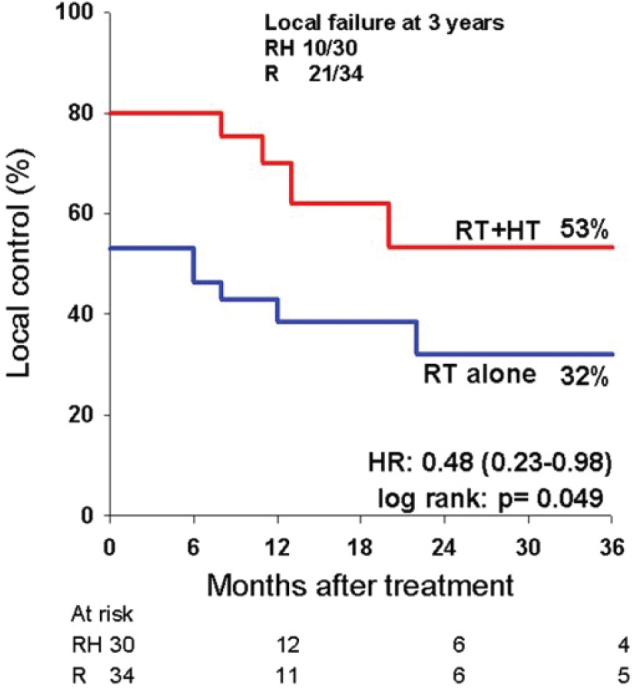
Local control in neck nodes after treatment with radiation alone or combined with hyperthermia.

The persistent local control was evaluated using the 3-year local control rate as endpoint. As seen in Figure 2, the difference between RT alone and RT+HT was maintained over time with 3-year values for local control of 32% vs. 53%, respectively (HR: 0.48 [0.23–0.98], *p* = 0.049). The univariate analysis (Table 3) also revealed that the response in recurrent tumors was poorer than in primary sites, whereas no significant difference in outcome as a function of nodal volume or N-strata was observed.

**Table 3:** Univariate analysis of initial complete response and 3-years local control as a function of treatment, prognostic or stratification group.

Group	Tumors	CR	LC
	No.	%	%
All tumors	64	66	42
Treatment			
RT	34	53*	32*
RT+HT	30	80*	53*
Volume			
< 10 cm^3^	21	67	39
10–25 cm^3^	22	63	47
> 25 cm^3^	21	67	42
Primary tumor	51	73*	51*
Recurrent tumor	13	38*	0*
Primary T-site			
Oral cavity	13	9	35
Larynx	6	83	83
Pharynx	41	63	41
Other	4	50	0

CR: complete response; LC: local control.

* *P* < 0.05.

### NED, disease-free and overall survival

Since only the N-site but not the primary T-site tumor was included in the evaluation, it was not meaningful to evaluate the outcome in form of NED and survival. This was further stressed by the fact that 10 patients had two nodal sites treated, both randomized to each of the 2 treatment arms. Thus, evaluation of patient related outcome cannot directly be linked with the treatment given to the individual neck node.

Considering that, and excluding the 10 patients who had multiple nodes treated with and without HT, 44 patients were left for analysis. Of these, 9 were alive without evidence of disease (6 in the RT+HT arm), and one patient (RT+HT) died disease-free from other causes. The remaining 34 patients all died from the HNSCC in question, 22 from T-site failure alone or jointly with other failure sites, (9 RT+HT); 6 patients (one RT+HT) died from distant metastases alone, and only 6 patients died from uncontrolled neck failure alone (4 RT+HT).

### Compliance and complications

The compliance to radiation was good, and 61 (95%) of the patients received the planned treatment (2 patients died during treatment). The acute and late radiation toxicity was acceptable. An acute moderate/severe radiation reaction was found in 17% and 19% of the patients treated with RT and RT+HT, respectively. The corresponding values for moderate/severe late effects were 13% and 20%, respectively. None of the values differed significantly between the two treatment arms.

HT was in general well-accepted, and the patients received between 1 and 7 (median 5) weekly treatments. In 22% of the treatments, no pain nor discomfort were found. Slight pain was observed in 40% of the heat sessions, moderate pain in 32%, and in 6% of the treatments the pain was so severe that the treatment was interrupted or stopped.

### Thermal parameters and quality of HT

The hyperthermic treatment was performed with electromagnetic heating with frequencies ranging from 144 to 915 MHz. Detailed reporting of temperature and heating details was achieved from 29 (97%) of the heated patients. Thermometry was performed as described in the ESHO quality assurance guidelines with thermocouples or thermistors. The number of temperature points in tumors range from 1to 8(median 3), and the number of points in surrounding normal tissue range from 0 to 9 (median 3).

In 119 of the 164 heat treatments (71%), in which some or all quality assurance data were available, the planned heating time was completed. The information available for each heat treatment were the minimal (Tmin) and the maximal (Tmax) heating time estimated as CEM43. In the normal tissue the maximal temperature was recorded. Tumor temperature data were achievable from 164 individual treatments, and normal tissue data were recorded in 159 treatments. The remaining data were not available either due to technical failure or loss of reporting.

**Figure 3:**
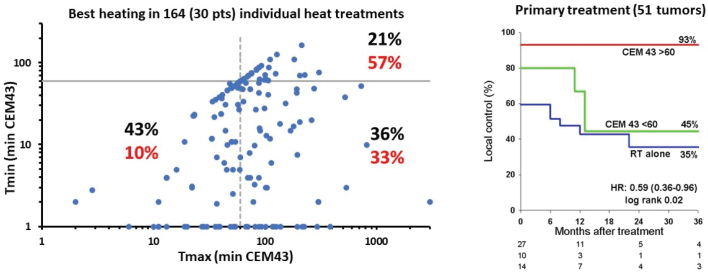
(Left) Relationship between the Tmax and Tmin in 164 heat treatments (in 30 patients). The dashed lines indicate the number of sufficient and insufficient heat treatments by separating between treatments above or below 60 min CEM43. Black numbers are data from all treatments. Red numbers indicate best heating in individual patients. (Right) Relationship between local control and heating quality (Above or below 60 min CEM43 or RT only) in 51 nodes given primary treatment. RT: radiotherapy; CEM43: cumulative equivalent minutes at 43°C.

Figure 3 shows an overview of the achieved temperatures and their mutual relationship. In 57% of the treatments the maximal tumor temperature (Tmax) reached the planned level (≥ 60 min CEM43). However, only in 21% of the heat sessions were the minimal measured tumor temperature above that level, Thus, most of the heat sessions did not obtain the planned HT QA target. Similarly, when evaluating the best heating obtained in each of the 30 nodes randomized to HT, did 57% of the recorded patients receive at least one heat session fulfilling the quality requirements. The normal tissue was in general kept at the low temperature level, and only 3% of the treatments yielded an CEM43 above 60 min, indicating a sufficient skin cooling.

An analysis of the importance of heating quality for the outcome of treatment was performed in the tumors in which sufficient temperature data were available. This included the 30 evaluable heated neck nodes, which were analyzed together with the 34 nodes treated with radiation alone. Sixteen nodes receiving at least one heat session with sufficient minimal heating had a persistent local control rate of 64%, compared to 39% in the 14 lymph nodes receiving insufficient heating, and 32% in the nodes treated with RT alone. This was especially found in the primary treated tumors (HR: 0.36 [0.16–0.80], *p* = 0.049), which together with CEM43 > 60 min (HR: 0.59 [0.36–0.96], *p* = 0.02) were the only parameters of significance for outcome when subjected to multivariate analysis. This is also illustrated in Figure 3 right, which shows a clear correlation between local control and CEM43 among the 51 primary treated tumors.

## Discussion

Despite its premature completion, the ESHO 2–85 trial is the largest randomized study evaluating the effect of adjuvant HT in association with a full course of RT in HNSCC neck nodes. The study demonstrates that HT significantly enhances the effect of radiation, both when evaluated as complete response and persistent local control. The benefit of the combined treatment confirmed the expected thermal enhancement in the order of 1.5, in line with the described experience from uncontrolled studies, and the hypothesis behind the study [5, 7]. Thus, the trial substantiated that such enhancement can be achieved with a weekly heat treatment. The benefit was obtained without enhancing radiation-related morbidity, and with tolerable heat related side effects.

The nature of the study where only a part of the tumor burden was given the treatment to be investigated made the trial a study of proof of principle, rather than a trial that significantly influenced the patient’s outcome of a given therapy. This may have influenced the participation in the study, which was stopped prematurely due to poor recruitment. Nevertheless, the number of evaluable neck-nodes were of the same magnitude or larger than in most previously reported prospective studies [20, 21, 24, 25], which make it a relevant contribution to the efficacy of HT in RT of HNSCC.

The previous clinical experience has pointed to tumors with larger volumes as the most sensitive to hyperthermic modification [4, 9, 20, 21]. Consequently, we have stratified the neck nodes to below or above 3 cm in the longest diameter, but we were unable to identify an independent significant influence on the outcome, probably due to the low number of tumors included.

Almost all patients achieved the planned radiotherapeutic treatment, but the heating quality was heterogeneous as also seen in other clinical trials with HT [10, 15, 18, 20]. Thus, only 21% of the heat treatments were in accordance with the protocol requirement, and only 57% of the tumors randomized to HT achieved at least one heat session that delivered the required CEM43 > 60 min to the entire tumor, which in turn resulted in a better outcome than the less or not heated nodes (Figure 3). Overall, the quality of the heating was at least of the same magnitude as that described in previous studies where similar parameters have been recorded [15, 18].

Many thermal parameters and characteristics have been suggested to be of prognostic value, but the outcome of the current protocol gives additional support to our previous experience, that the thermal parameter of pivotal importance is the ability to secure a sufficient minimal temperature in at least one heat session [5, 7, 9, 10, 11, 15, 18, 20], thereby taking advantage of the hyperthermic cytotoxicity but avoiding thermotolerance [5, 26].

The observation of a better response in primary than in recurrent tumors was in line with the randomized RTOG 81–4 trial [11] and may be linked with the general radioresistance seen in recurrent HNSCC [33]. The complications to treatment were minor and acceptable and despite most heat treatments being given within half an hour after radiation, this did not result in any notable enhanced radiation damage. This is similar to other studies [18, 21, 22, 25] and may partly be due to the use of active skin cooling in many cases.

The current rapport brings the initial European collaborative trial activity to an end. In addition to the current ESHO 2–85 study on advanced neck nodes, HT has also been found to significantly enhance the effect of RT in the treatment of locally advanced breast carcinoma, recurrent breast carcinoma in the chestwall, malignant melanoma, and in advanced pelvic tumors [14–18].

The overall implication of the ESHO 2–85 study is the confirmation of HT as a tool to enhance the effect of ionizing irradiation, and thereby gives it a place in situations where an acceptable local control with RT alone cannot be achieved, such as in the treatment of recurring tumors and/or in previously irradiated sites. The results further supported the high-level proof-of-concept for the effectiveness of HT when added to RT. Pending more advanced HT technologies, this combination has the potential to recieve more attention in the future as part of multidisciplinary treatment strategies.

## Conclusion

Overall univariate and multivariate analysis both showed that adjuvant HT significantly improved local control in the neck nodes when applied weekly with conventional fractionated RT. The effect of adjuvant HT was related to the extent and quality of the heating achieved. HT did not enhance the early or late radiation morbidity. Compliance and tolerance to HT was good, but half of the tumors did not receive the prescribed minimal heat dose. The study confirmed the underlaying hypothesis of a clinically useful thermal enhancement of ionizing irradiation. Despite the long history and incompleteness of the trial, it supports the need for continuous exploration of the potential of hyperthermic oncology [1, 34].

## Data availability

Non-person identified data can be obtained from the corresponding author upon reasonable request and subjected to approval from the relevant ethics committee.

## Supplementary Material

ESHO 2–85. Hyperthermia as an adjuvant to radiation therapy in the treatment of advanced neck nodes: A randomized multicenter study by the European Society for Hyperthermic Oncology
