# Supplementary material for: ESHO 2–85. Hyperthermia as an adjuvant to radiation therapy in the treatment of advanced neck nodes: A randomized multicenter study by the European Society for Hyperthermic Oncology
Source: Acta Oncol. 2024 Dec 12;63:41035. doi: 10.2340/1651-226X.2024.41035 (PMC11656068; doi:10.2340/1651-226X.2024.41035)
Supplement: ESHO 2–85. Hyperthermia as an adjuvant to radiation therapy in the treatment of advanced neck nodes: A randomized multicenter study by the European Society for Hyperthermic Oncology [file AO-63-41035-s1.pdf]

Supplementary material has been published as submitted. It has not been copyedited, or typeset by Acta Oncologica

EUROPEAN SOCIETY FOR HYPERTHERMIC ONCOLOGY

# E. S. H. O.

## PROTOCOL 2-85

### HYPERTHERMIA AS AN ADJUVANT TO RADIOTHERAPY IN THE TREATMENT OF ADVANCED NECK NODES

### - A RANDOMIZED MULTICENTER STUDY

THIRD EDITION
SEPTEMBER 1988

E.S.H.O. Protocol 2-85

# Hyperthermia as an adjuvant to radiotherapy in the treatment of advanced neck nodes

**A randomized multicenter study**

Fourth edition
December 1986

---

**Study coordinator:**

Jens Overgaard, M.D.
The Danish Cancer Society
Department of Experimental Clinical Oncology
Radiumstationen
DK-8000 Aarhus C
Denmark
Phone: +45 86 12 36 45
Telefax: + 45 86 19 71 09

## BACKGROUND

It is a long known and well established fact that hyperthermia is capable of enhancing the effect of ionizing radiation. Abundant information on both biological, technical, and clinical aspects of such combined treatment has been gained in recent years, all pointing towards the feasibility of applying hyperthermia as a modality which may be useful in treating especially large superficial tumours (3,4,5,7,8,19,20,23,24,26,32,33).

### Principles of interaction between heat and radiation

The biological interaction between heat and radiation consists of at least two different principal types of interaction (4,5,7,18,19,20,23,24,33).

Firstly, heat has a direct cytotoxic effect. Although the heat sensitivity varies among the different cells and tissues, the intrinsic heat sensitivity does not seem to be higher in malignant cells than in their normal counterparts. However, the hyperthermic cytotoxicity is strongly enhanced by certain environmental conditions, and cells in areas characterized by nutritional deprivation, chronic hypoxia, and increased acidity, which are typical of the poorly vascularized parts of solid tumours, are considerably more heat sensitive than cells in a normal environment. Thus, a moderate heat treatment, which can be tolerated by well-vascularized normal tissues, is found to destroy a large proportion of the cells in many solid tumours. Furthermore, heat itself may enhance such environmental conditions in tumours by reducing the blood flow (32). The fact that such heat sensitive, chronically hypoxic cells are also the most radioresistant, may indirectly influence the response to a combined heat and radiation treatment, since a smaller radiation dose may be adequate to control the remaining oxygenated cells (21,22).

Secondly, hyperthermia has a radiosensitizing effect which is expressed as a direct sensitization of the radiation damage. The mechanism hereof is complex and enrolls both a direct radiosensitization, a decreased repair of sublethal and potentially lethal radiation damage, and an enhanced killing of cells in radioresistant phases of the cell cycle. The oxygen enhancement ratio, however, seems not to be decreased by this combined treatment. In contrast to the cytotoxic effect is the hyperthermic

radiosensitization apparently present to the same extent in both malignant and benign tissues.

Whereas the cytotoxic destruction of radioresistant tumour cells bears no time-relationship to the radiation treatment, the heat induced radiosensitization is strongly dependent on the sequence and interval between the two modalities. Maximal sensitization is achieved by a simultaneous application of the two treatment components, and any separation between the two modalities decreases the radiosensitizing effect which normally disappears with intervals longer than 3-4 hours (7,20,27).

Both the magnitude of the hyperthermic radiosensitization and the cytotoxic effect augment with increasing heat damage (i.e. combination of temperature and heating time). The effect expressed as the thermal enhancement ratio (TER) is most prominent in the radiosensitizing effect. The cytotoxic effect is smaller, but when a moderate heat treatment is given, this effect is selective for the tumour since cells in a normal environment seem not to suffer significant damage. This dual mechanism of interaction between hyperthermia and radiation gives us the following rationales for applying hyperthermia as an adjuvant to radiotherapy:

1) to increase the biological effect of a given radiation dose (hyperthermic radiosensitization)
2) to destroy radioresistant tumour cells (hyperthermic cytotoxicity)

The choice between the two rationales depends on whether or not it will be possible to heat the tumour area selectively or at least to a significantly higher extent than the surrounding normal tissue (23,24). A simultaneous heat and radiation application should be aimed at if selective tumour heating can be achieved. However, if normal tissue is part of the heated area hyperthermic radiosensitization should be avoided. This can be obtained by applying hyperthermia 3-4 hours after radiation (sequential treatment). Although this results in a lower thermal enhancement ratio it will occur only in tumours where radioresistant cells are selectively destroyed due to their deprived environment (23,24).

Thermotolerance and fractionated treatment

A clinical application of hyperthermia together with radiotherapy is naturally most likely to be given in fractionated sche-

dules. Recent studies have shown that this may process an important problem related to the phenomenon known as thermotolerance (i.e. a temporary heat resistance following a prior heat treatment) (6,17,28). The presence of thermotolerance has been found to reduce both the hyperthermic radiosensitization and the effect of heat when given sequentially with radiation as a cytotoxic agent (12,15,16,29). Unfortunately there is a considerable variation in the kinetics and the development of thermotolerance between different tissues, and it is currently not possible to predict how thermotolerance will develop in a given tumour or normal tissue (5,6,17,29). However, both the magnitude and the kinetics of thermotolerance appear to depend on the heat damage induced by the prime heat treatment (13,15,29). Thus, in a given tissue thermotolerance will develop later, but will reach a higher maximum if the prime heat treatment is large. This further complicates the problem, because if a homogeneous temperature cannot be applied to a given tissue, different tumours will develop thermotolerance with different kinetic patterns. Therefore, at the time of a subsequent heat treatment the various heterogenously heated areas will express different heat sensitivity as a function of the extent of the thermotolerance present. The best way to properly overcome the problems of thermotolerance will be to apply hyperthermia as a single, or a few large, heat fraction(s) given with an interval which hopefully is long enough to allow thermotolerance both to develop and to decay before the next hyperthermic treatment is given. Such a heat treatment principle could easily be combined with conventional radiation if the sequential (cytotoxic) treatment principle is applied. With simultaneously fractioned treatment the problem is more complex since an optimal utilisation of the radiosensitizing principle requires both a lack of thermotolerance and a concomitant heat treatment with all radiation fractions (22,29). In order to avoid the influence of thermotolerance, radiation must also be given with relatively large intervals, e.g. once or twice a week. Therefore, when using simultaneous treatment we may have a situation where the schedule which could be best from the heating point of view holds a less than optimal radiation treatment and vice versa.

Clinical experience

The clinical experience with combined heat and radiation has rapidly accumulated in recent years, and significant information has been gained about the potential of using hyperthermia as an adjuvant to radiotherapy (8,24,25,30). Table 1 shows a series of studies where comparable lesions have been treated with either radiation alone or combined heat and radiation. Although the number and size of heat and radiation fractions as well as the sequence and interval between the two modalities differ among these studies, a remarkable heat improvement of the radiation response has been uniformly achieved.

**EFFECT OF ADJUVANT HYPERTHERMIA ON THE RADIATION RESPONSE**

| STUDY | PATIENTS or TUMOURS | FREQUENCY OF COMPLETE RESPONSE | |
|---|---|---|---|
| | | Radiation alone | Radiation+Heat |
| Arcangeli et al. | 163 | 38% | 74% |
| Perez et al. | 154 | 41% | 69% |
| Overgaard | 101 | 39% | 62% |
| U et al. | 14 | 14% | 86% |
| Kim et al. | 238 | 39% | 72% |
| Bide et al. | 76 | 0% | 7% |
| Hiraoka et al. | 33 | 25% | 71% |
| Kochegarov et al. | 161 | 16% | 63% |
| Lindholm et al. | 85 | 25% | 46% |
| Corry et al. | 34 | 0% | 62% |
| Scott et al. | 62 | 39% | 87% |
| Li et al. | 124 | 29% | 54% |
| van der Zee et al. | 71 | 5% | 27% |
| Steeves et al. | 75 | 23% | 61% |
| Dunlop et al. | 86 | 50% | 60% |
| Gonzalez et al. | 46 | 33% | 50% |
| Valdagni et al. | 78 | 36% | 73% |
| Bey et al. | 45 | 9% | 42% |

(from ref. 37).

Also site and tumour specific data can now be evaluated. Especially three tumour types have been the subject of combined heat and radiation therapy (1,2,8,9,10,11,22,25,26,27,30,31,34, 35,36). This includes advanced neck nodes, recurrent or advanced (T3-T4) breast tumours and malignant melanoma.

With our present knowledge, there seems to be sufficient justification for applying adjuvant hyperthermia in the primary

treatment of cancer with the prime purpose of local control of bulky primary lesions. Advanced head and neck cancer is a tumour which seems to fulfil the requirement for a site specific phase III clinical trial. However, studies on this tissue type may have to be limited to the lymph nodes as the primary tumour may be situated too deeply and be beyond sufficient heating. Such nodes are frequently treated with primary radiation, though unfortunately often with limited success.

Accumulating evidence from recent phase II clinical trials indicates that a significant improvement in local control is likely to be gained without increasing normal tissue morbidity (1,2,9,25). Thus, a dose-response analysis of available data indicated a thermal enhancement of approximately 1.5 when adjuvant hyperthermia was applied to radiotherapy (figure 1). Although crude, such dose-response data give a hint as to the magnitude of the expected gain in prospective controlled clinical trial.

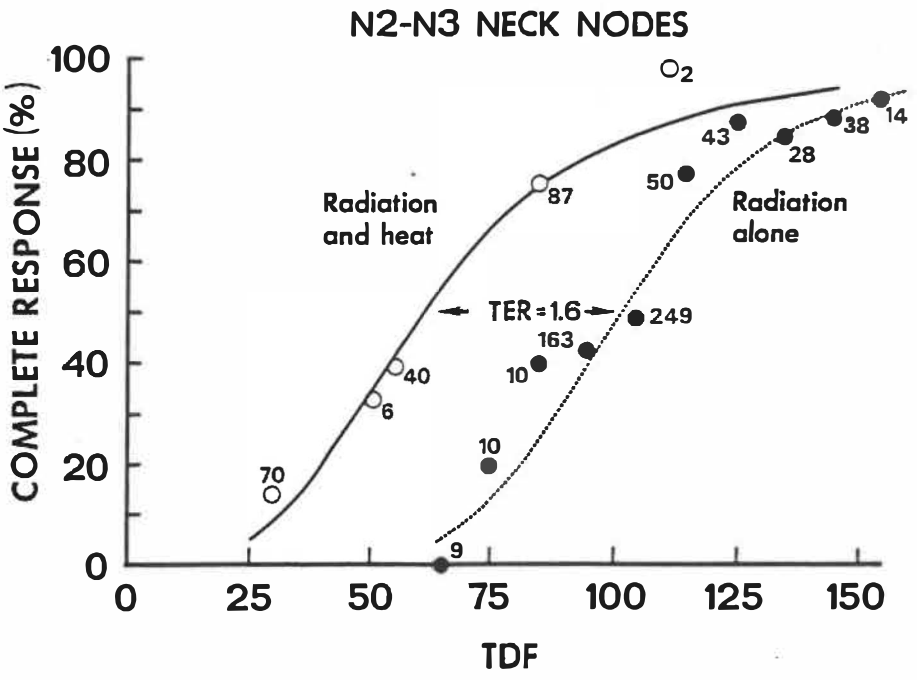


Figure 1
An estimate of the dose response relationship for advanced neck nodes treated with radiation alone or combined radiation and hyperthermia. Data from literature and unpublished observations

Furthermore, neck nodes are normally rather easy to heat due to their superficial site and evaluation is also easy.

When dealing with primary treatment of malignant lesions in patients with a relatively long life expectancy, the combined treatment must be at least as successful as radiotherapy alone, and the risk of especially enhanced late effects must be minimal. This reduces the possibility for altering the radiation treatment schedule. We must consequently add hyperthermia to irradiation treatment schedules already known to be as effective as possible. The treatment principle should therefore be based on conventional radiotherapy, using a standard fractionation schedule with daily radiation of approximately 2 Gy per fraction, investigating whether additional heat treatment given once or twice a week improves the tumour destructive effect. The argument has been discussed in detail, especially with regard to the development of thermotolerance and the interval between the hyperthermic fractions (6,22,24,29). The potential advantage of hyperthermia in such a schedule will be its cytotoxic rather than its radiosensitizing effect, as it would otherwise have to be given in connection with all radiation fractions (22,29).

If normal tissue heating can be avoided, heat treatment should be given immediately after radiation, as some degree of radiosensitization may add to the tumour destructive effect. Otherwise, an interval of approximately 3 to 4 hours should be allowed between radiation and hyperthermia to avoid sensitization of the radiation response in the normal tissue (24).

## OBJECTIVES

This is a multicenter randomized and balanced study with the object:

1) to assess the efficacy of local hyperthermia given as an adjuvant to primary radiotherapy of advanced neck nodes in patients with primary squamous cell carcinoma of the head and neck.
2) to evaluate survival, the rate of tumour regression, local control and frequency of recurrence, if any, of malignancy both within and outside the treated field.

3) to assess early and late tolerance of normal tissues subjected to radiation alone or radiation combined with adjuvant hyperthermia.

4) to compare the feasibility of various clinical heating techniques and thermometry in measuring and achieving a local homogeneous heating in metastatic neck nodes.

**PATIENT SELECTION**

Patients with advanced squamous cell carcinoma of the head and neck who are candidates for primary radiotherapy should be included provided they fulfill the following criteria:

1) the patients should have metastatic neck nodes clinically classified as N1, N2, or N3 (UICC classification 1982).
2) the primary tumour or nodes should be proven to be an invasive squamous cell carcinoma by histopathological (fine needle) examination. Excisional biopsi not accepted.
3) the nodes should be well defined and measurable in at least 2 dimensions.
4) the patients should have a life expectancy greater than 6 months.
5) the patients must not have been subjected to other cancer therapy, especially chemotherapy, for 4 weeks prior to or concurrently with protocol therapy. The area must not previously have been treated with radiotherapy.
6) frequent follow-up should be possible.
7) the tumour should be considered feasible for heating with the available equipment.
8) the patients should accept participation in the study.

**PRETREATMENT EVALUATION**

Routine clinical examinations should be performed prior to treatment.

The tumour should be measured in at least 2, if possible, 3 diameters using a vernier caliper at the times required by the protocol. An initial estimate should be made of the depth from the skin surface to the superficial and deep margin of the tumour. The

tumour should be drawn on an anatomical diagram. CT-scanning or ultra-sound is recommended to give more objective measurements of tumour size and depth.

Colour-photographs of the fields, preferably with the temperature probes in situ, and lesions using a standard focus distance, standard background, standard illumination, and a standard film should be obtained. Centimeter scale should be used on each photograph.

Prior treatment should be specified.

Laboratory examinations should include whole blood count, and hemoglobin, but otherwise no laboratory examinations are mandatory.

Otherwise the patients should be evaluated according to the normal department policy.

## TRIAL DESIGN

Patients fulfilling the above criteria are stratified according to institution and size of nodes (≤ 3 cm / > 3 cm), and randomized to one of the following schedules:

1) Conventional radiotherapy alone (60 Gy tumour dose to gross tumour, if needed followed by a boost up to 10 Gy through reduced portals to the residual tumour).
   The radiotherapy should be given in daily fractions of approximately 2 Gy five times per week.
2) Conventional radiation administered as above. Once weekly the 5 first weeks the radiation treatment should be followed within 30 minutes by hyperthermia. The heat session should aim for a minimal tumour treatment temperature treatment of 43.0 °C in 60 minutes allowing additional time for induction and cooling of the tumour.

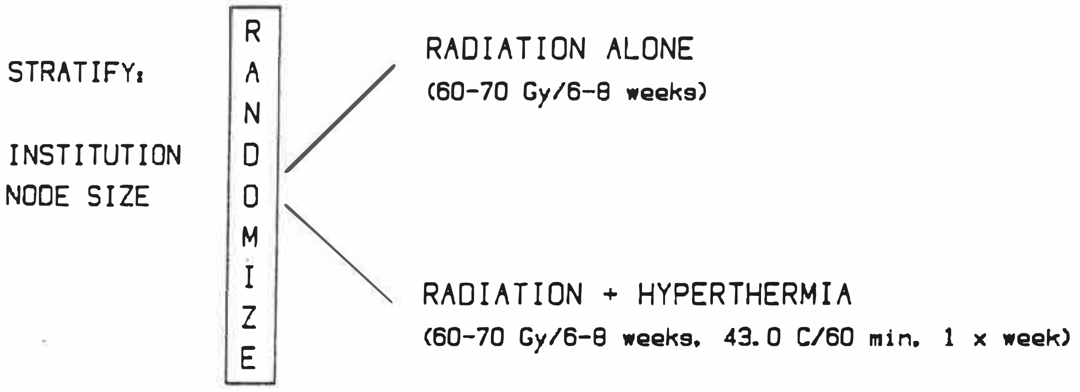

All patients should be seen at 2 and 4 weeks after completion of therapy, then monthly for the next 3 months, every 3 months the rest of the first year, every four months during the second and third year, and every six months thereafter. In case of local failure or recurrence this schedule can be simplified.

At each follow-up normal tissue damage and local tumour response should be recorded. If the tumour or disease in general does not respond to treatment, additional treatment can be given.

The end point of the study is persistent local control, acute and late normal tissue damage. Since variating tumour size and doses exist does the data allow dose-response curves to be made, and on this basis a thermal enhancement ratio is likely to be achieved.

## TREATMENT

### Radiotherapy

Radiotherapy should be given as specified in ICRU-29.

Radiation should be applied using electrons or photons through one or multiple portals according to the departments' general policy. The treatment should be selected to provide not less than a 90% iso-dose to the macroscopic tumour. The skin dose should be specified (TLD measurements are recommended). Dose-rates should be between 0.5 and 5 Gy/ minute. The given tumour dose to tumour areas (T and N position) should be 60 Gy. A subsequent boost up to 10 Gy through reduced portals may be added to the residual primary tumour and nodes. The primary tumour should receive at least the same dose as the nodes. The given tumour dose to potential microscopic envolved areas should be at least 50 Gy.

Radiation should be delivered in daily fractions of 2 Gy, 5 fractions per week.

In patients with multiple nodes receiving both radiotherapy alone and combined treatment all nodes must receive identical radiotherapy. The planned radiotherapy schedule must be specified prior to randomization.

### Hyperthermia

There is no limitation to the equipment used for hyperthermic treatment, except that it should be likely to provide a tumour

temperature of 43.0 °C. Active skin cooling may be used. The heat treatment should be applied once weekly after radiation treatment. The treatment should start as soon as possible after the irradiation and not more than 30 minutes after. At each session heat treatment should aim for 60 minutes at 43.0 °C. The initial time (up to 10 minutes) needed to increase the temperature to this point as well as the cooling time should not be included in the heating time. An effort should be made to avoid heating the normal tissue. If this cannot be avoided, the investigator should be aware of the possible risk of enhancing radiation damage, and an interval larger than 3 hours between radiation and hyperthermia may be considered for subsequent treatments, in order to reduce this risk.

The heat treatment should continue for the first 5 weeks to a total of 5 treatments if gross tumour is still present.

At completion of each heat-treatment the temperature should continue to be recorded until below 40 °C. Such thermal washout-curves will create a data-base for further studies of the blood-flow and heating patterns in tumours.

The use of local anaestetics during heating should be avoided and patient's sensation of pain should be noted, since it may be an important reference to normal tissue damage.

GUIDELINE FOR TEMPERATURE MEASUREMENT POINTS

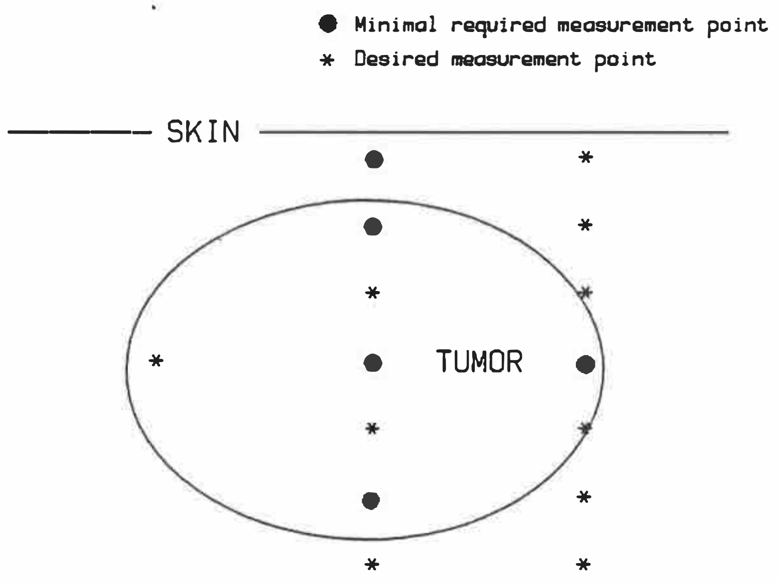

Multi-point temperature recording should be obtained at regular intervals (< 2 minutes). Temperature should be recorded at specified points in normal tissue and tumour (see figure). Also cooling water (water bag) temperature should be recorded. It is important to obtain as many temperature measuring points as possible and the use of multi-point temperature sensors is strongly recommended. If possible, the temperature measurement points should be correlated with CT-scan or ultrasound. The reference temperature is the lowest measured tumour temperature; the other temperatures should only be recorded.

Thermal dose data should be recorded, if possible, as equivalent heating time at 43.0 °C.

Each participating center should keep a file of the applied radiotherapy, heating technique, and temperature measurements, and a technical sub-committee will evaluate the quality of the heat treatment at the individual centers. Since the study also aimed at analyzing the feasibility of different heating techniques, it is important to register as many parameters as possible.

## EVALUATION OF RESPONSE

The evaluation of the objective response should be based on clinical, ultrasound, CT-scanning or x-ray examination using the guidelines laid down in WHO recommendations (14). All visible responses should be documented by colour photographs.

### Evaluation during treatment

Tumour response and measurements should be recorded weekly according to the above-mentioned guidelines.

The skin response should be evaluated and the acute peak reactions should be recorded.

Specific thermal damage to normal tissue (e.g. thermal blisters) should be noted.

### Post-treatment evaluation

It is recommended that the patients are followed by the same clinicians, if possible, in order to avoid individual variations in assessment.

The patients should be seen at 2 and 4 weeks after treatment, then monthly for the next 3 months, and every three months

for the rest of the first year, every fourth month during the second and third year, and every six months thereafter (may be simplified in case of local failure). Yearly ultrasound or CT-scanning is recommended.

Information to be recorded at each follow-up should include tumour measurement as indicated before, skin scoring for acute or late radiation and/or hyperthermic damage. The data should be documented by colour photographs, if needed. CT-scanning or ultrasound should be repeated as frequent as possible as long as tumour tissue is present. Local failure should be histopathologically (fine needle) confirmed.

At time of death the final evaluation of the patient should take place, and if possible autopsy material from the treated area should be collected.

Withdrawal from study

If the patient's condition makes completion of treatment impractical and the protocol cannot be followed, the alternative treatment should be recorded. Follow-up of the patient should continue, if possible.

If the patients after treatment show evidence of recurrent or progressive disease elsewhere, additional treatment, if required, should be given in the most optimal form. The necessary step taken should be reported, and the patient should continue the follow-up as planned. **Such patients should NOT be withdrawn from the study.**

## REGISTRATION AND RANDOMIZATION OF PATIENTS

Central randomization is performed by the Institute of Cancer Research, Aarhus, Denmark. To register a patient **call** +45 86 120645, monday-friday, from 8 am to 3 pm, GMT.

The following information will be requested:

1) protocol number
2) name of institution and investigator
3) patient identification.
4) number of eligible nodes (potential heating fields)

5) N-classification
6) size of node(s)
7) each field should be given a number for randomization purpose
8) planned radiation treatment
9) performance status

Upon this information a treatment schedule will be assigned to each field as follows:

| | |
|---|---|
| Patients with 1 field/node: | randomisation to radiation alone or combined treatment |
| Patients with 2 fields/nodes: | one will receive radiation alone, the other combined treatment |
| Patients with 3 or more fields/nodes: | at least one field will be randomized to each treatment |

Registration of patients and the response to treatment should be mailed to the study coordinator, who will perform the data collection.

| | |
|---|---|
| 1) On study form: | should be completed at time of randomization. The form includes patient identification, pre-treatment evaluation, and the planned treatment. |
| 2) Treatment form: | a summary of the treatment and immediate follow-up should be completed one month after treatment. |
| 3) Follow-up forms: | should be completed at all visits. |
| 4) Death form: | should be used at time of death of the patient. |

## STATISTICAL CONSIDERATIONS

The known dose-response relationship for both radiation alone and combined radiation and hyperthermia treatment of advanced neck nodes indicates that a treatment gain in the order of 30% improvement in local control is to be expected. The trial will be closed after intake of 120 evaluable patients (> 120 nodes). If the true frequency of any event is changed by 30%, e.g. from 50% to 80%, the likelihood that a significant difference is observed ($P<0.05$) is more than 90%.

In the final analysis of the trial all frequencies will be calculated by use of the life-tabel method, and the statistics estimated by use of the log-rank test. Dose-response relationship will be estimated by logit analysis. In addition to the stratification groups, the final analysis will include an evaluation according to other prognostic parameters (N-classification and localization of primary tumour).

## ETHICAL CONSIDERATIONS

The study is designed according to the requirements laid down by the Helsinki Declaration II. After careful considerations of the predictabel risks it is the responsible investigator´s judgement that the project does not present etical problems. The patient will be given information on the aims, methods, anticipated benefits and potential hazards. Only patients randomized to receive hyperthermia will be given information about this part of the treatment.

The protocol should be adapted to and approved by all relevant national or local etical committees.

## ADMINISTRATION RESPONSIBILITIES

The protocol is a joint effort among the investigators and coordinated by the European Society for Hyperthermic Oncology. The clinical and technical data will be evaluated by the study coordinator in collaboration with the principal investigators from the participating institutions. The group will meet at least once a year during the annual meeting of the European Society for Hyperthermic Oncology.

PUBLICATION GUIDELINES

1) Each publication based on this material will originate from the European Society for Hyperthermic Oncology (E.S.H.O.), and will name all of the partcipating institutions.

2) Authors of the publication(s) should be two members from each participating institution which includes at least 5 evaluable patients in the trial, active members of the clinical committee and the statistician are co-authors of the publication(s). In addition, may a person who has contributed significantly to the study, e.g. with quality assurance, be included as author.

3) Each participating institution can use material for regional /national local information in lecture form by mentioning the name of the other participating institutions.

4) Data (papers or published abstracts) related to the aims of the study (outlined in Objectives) cannot be published separately by a participating institution, unless agreed upon by the Clinical Committee. Locally initiated spin-off projects can be published by the respective responsible project members. All publications must mention that the material originated from the E.S.H.O. trial, and must not be published prior to the whole material.

5) Initiatives for publication will be taken by the Clinical Committee. All manuscripts will be circulated for comments by the participating institutions before publication.

6) All questions about publication policy should be addressed to the E.S.H.O. Clinical Committee before any manuscript is submitted for publication. Their decision can be appealed to the Board of E.S.H.O. which has the final decision.

## STUDY COORDINATOR

Jens Overgaard, M.D.
The Danish Cancer Society
The Department of Experimental Clinical Oncology
Radiumstationen
DK - 8000 Aarhus C
Denmark
telephone: +45 86 12 06 45
telefax: +45 86 19 71 09

## STATISTICIAN

Søren M. Bentzen, Ph.D.

## MEMBERS OF E.S.H.O. CLINICAL COMMITTEE

G. Arcangeli, Rome
J.-M. Cosset, Paris
D. Gonzalez Gonzalez, Amsterdam
J. Overgaard, Aarhus (Chairman)

## REFERENCES


1. Arcangeli, G., Barni, E., Cividalli, A., Mauro, F., Morelli, D., Nervi, C., Spano, M., and Tabocchini, A. (1980): Effectiveness of microwaved hyperthermia combined with ionizing radiation: Clinical results on neck node metastases. Int. J. Radiation Oncology Biol. Phys. 6: 143-148.

2. Arcangeli, G., Nervi, C., Cividalli, A., Lovisolo, G.A., and Mauro, F. (1985): The clinical use of experimental parameters to evaluate the response to combined heat (HT) and radiation (RT). In: Hyperthermic Oncology 1984, vol. 1, edited by J. Overgaard, pp. 329-332, Taylor & Francis, London and Philadelphia.

3. Dethlefsen, L.A. and Dewey, W.C.(eds) (1982): Proceedings of the Third International Symposium on Cancer Therapy by Hyperthermia, Drugs and Radiation. Natl Cancer Inst Monogr 61.

4. Dewey, W.C., Freeman, M.L., Raaphorst, G.P., Clark, E.P., Wong, R.S.L., Highfield, D.P., Spiro, I.J., Tamasovic, S.P., Denman, D.L., and Coss, R.A. (1980): Cell biology of hyperthermia and radiation. In: Radiation Biology in Cancer Research, edited by R.E. Meyn and H.R. Withers, pp. 589-621. Raven Press, New York.

5. Field, S.B. (1983): Cellular and tissue effect of hyperthermia and radiation. In: The Biological Basis of Radiotherapy, edited by. G.G. Steel et al. pp. 287-303. Elsevier, Amsterdam, New York, Oxford.

6. Field, S.B. (1984): Clinical Implications of Thermotolerance. In: Hyperthermic Oncology 1984, vol 2, edited by J. Overgaard, pp. 235-244. Taylor & Francis, London and Philadelphia.

7. Field, S.B. and Bleehen, N.M. (1979): Hyperthermia in the treatment of cancer. Cancer Treat Rev 6:63-94.

8. Hahn, G.M. (1982): Hyperthermia and Cancer (Plenum Press, New York and London).

9. Hofman, P., Lagendijk, J.J.W., and Schipper, J.: The Combination of Radiotherapy with Hyperthermia in Protocolized Clinical Studies. In: Hyperthermic Oncology 1984, vol 1, edited by J. Overgaard, pp. 379-382. Taylor & Francis, London and Philadelphia.

10. Kim, J.H., Hahn, E.W., and Ahmed S.A. (1982): Combination Hyperthermia and Radiation Therapy for Malignant Melanoma. Cancer 50: 478-482

11. Kim, J.H., Hahn, E.W., S.A. Ahmed, and Kim Y.S.: Clinical Study of the Sequence of Combined Hyperthermia and Radiation Therapy of Malignant Melanoma. In: Hyperthermic Oncology 1984, vol 1, edited by J. Overgaard, pp 387-390. Taylor & Francis, London and Philadelphia.

12. Kamura, T., Nielsen, O.S., Overgaard, J., and Andersen, A. H. (1982): Development of thermotolerance during fractionated hyperthermia in a solid tumor in vivo. Cancer Res 42:1744-1748.

13. Law M. P., Coultas, P.G., and Field, S.B. (1979): Induced thermal resistance in the mouse ear. Br J Radiol 52:308-314.

14. Miller, A.B., Hoogstraten, B., Staquet, M., and Winkler, A. (1981): Reporting Results of Cancer Treatment. Cancer 47: 207-214.

15. Nielsen, O.S. and Overgaard, J. (1982): Importance of preheating temperature and time for the induction of thermotolerance in a solid tumour in vivo. Br J Cancer 46:894-903.

16. Nielsen O. S., Overgaard, J., and Kamura, T. (1983): Influence of thermotolerance on the interaction between hyperthermia and radiation in a solid tumour in vivo. Br J. Radiol 56:267-273.

17. Nielsen, O.S. (1984): Fractionated hyperthermia and thermotolerance. Danish Medical Bulletin 31:376-390.

18. Overgaard, J. (1977): Effect of hyperthermia on malignant cells in vivo. A review and hypothesis. Cancer 37:2637-2648.

19. Overgaard, J. (1978): The effect of local hyperthermia alone and in combination with radiation, on solid tumors. In: Cancer Therapy by Hyperthermia and Radiation, edited by C. Streffer et al. pp.49-61. Urban and Schwarzenberg, Baltimore, Munich.

20. Overgaard, J. (1980): Simultaneous and sequential hyperthermia and radiation treatment of an experimental tumor and its surrounding normal tissue in vivo. Int J Radiat Oncol Biol Phys 6:1507-1517.

21. Overgaard, J. (1981): Effect of hyperthermia on the hypoxic fraction in an experimental mammary carcinoma in vivo. Br J Radiol 54:245-249.

22. Overgaard, J. (1981): Fractionated radiation and hyperthermia Experimental and clinical studies. Cancer 48:1116-1123.

23. Overgaard, J. (1982): Influence of sequence and interval on the biological response to combined hyperthermia and radiation. Natl Cancer Inst Monogr 61:325-332.

24. Overgaard, J. (1983): Hyperthermic modification of the radiation response in solid tumors. In: Biological Bases and Clinical Implications of Tumor Radioresistance, edited by GH Fletcher et al. pp 337-352. Masson Publishing USA, New York.

25. Overgaard, J. (1985): Rationale and Problems in the Design of Clinical Studies. In: Hyperthermic Oncology 1984, vol. 2, edited by J. Overgaard, pp. 325-338. Taylor & Francis, London and Philadelphia.

26. Overgaard, J. (ed) (1985): Hyperthermic Oncology, vols. 1 & 2, Taylor & Francis, London and Philadelphia.

27. Overgaard, J. (1985): Hyperthermia and Radiation - an update of Biological and Clinical Experience. In: Proceedings of the XVI International Congress of Radiology, pp. 211-217.

28. Overgaard, J. and Overgaard, M. (1985): A clinical trial evaluating the effect of simultaneous or sequential radiation and hyperthermia in the treatment of malignant melanoma. In: Hyperthermic Oncology 1984, vol. 1, edited by J. Overgaard, pp. 383-386, Taylor & Francis, London and Philadelpia.

29. Overgaard, J. and Nielsen, O.S. (1983): The importance of thermotolerance for the clinical treatment with hyperthermia. Radiotherapy and Oncology 1:167-178.

30. Perez, C.A. and Meyer J.L. (1985): Clinical experience with localized hyperthermia and irradiation. In: Hyperthermic Oncology 1984, vol. 2, edited by J. Overgaard pp. 181-198. Taylor & Francis, London and Philadelphia.

31. Perez, C.A., Nussbaum G., Emami, B., and VonGerichten, D. (1983): Clinical Results of Irradiation Combined With Local Hyperthermia. Cancer 52: 1597-1603.

32. Storm, F.K. (ed.) (1983): Hyperthermia in Cancer Therapy (G.K. Hall Medical Publishers, Boston, Massachusetts).

33. Suit, H. and Gerweck, L.E. (1979): Potential for hyperthermia and radiation therapy. Cancer Res 39:2290-2298.

34. van der Zee, J., van Rhoon, G.C., Wike-Hooley, van den Berg, A.P., Reinhold, H.S.: Thermal Enhancement of Radiotherapy in Breast Carcinoma. In: Hyperthermic Oncology 1984, vol. 1, edited by J. Overgaard, pp. 345-348. Taylor & Francis, London and Philadelphia.

35. Gonzalez Gonzalez, D., van Dijk, J.D.P., Blank, L.E.C.M., and Rümke, Ph. (1986): Combined treatment with radiation and hyperthermia in metastatic malignant melanoma. Radiotherapy and Oncology 6: 105-113.

36. Perez, C.A., Kuske, R.R., Emami, B., Fineberg, B.: Irradiation alone or combined with hyperthermia in the treatment of recurrent carcinoma of the breast in the chest wall. A nonrandomized comparison. Int J Hyperthermia 2: 179-189.

37. Overgaard, J. (1986): The design of clinical trials in hyperthermic oncology. In: Physics and Technology in Hyperthermia. Edited by S.B. Field and C. Franconi. Elsevier. (in press).

Appendix I

## SCORING OF ACUTE AND LATE RADIATION DAMAGE

| | Erythema | Moist desquamation | Telangi-ectasia | Fibrosis |
|---|---|---|---|---|
| grade 0 | none | none | none | palpably not different from unexposed skin + subcutaneous tissue |
| grade 1 | mild | < 10% of field | < 1/cm$^2$ | just palpable increased density and firmness of exposed skin + subcutaneous tissue |
| grade 2 | moderate | $\geq$ 10% < 50% of field | $\geq$ 1< 4/cm$^2$ | definite increased density and firmness of exposed skin + subcutaneous tissue |
| grade 3 | severe with dry desquamation | $\geq$ 50% of field | $\geq$ 4/cm$^2$ | very marked density, retraction and fixation of exposed skin + subcutaneous tissue |

## Appendix II

**SCORING OF ACUTE HEAT DAMAGE** *

| | Blisters | Necrosis ** |
|---|---|---|
| grade 0 | none | none |
| grade 1 | mild | < 10% of field |
| grade 2 | moderate | ≥ 10% < 50% of field |
| grade 3 | severe with dry desquamation | ≥ 50% of field |

*) In heated field only
**) Necrosis in tumours penetrating the skin should not be included

## Appendix III

**PERFORMANCE STATUS**

| Grade | |
|---|---|
| 0 | Able to carry out all normal activity without restriction |
| 1 | Restricted in physically strenuous activity but ambulatory and able to carry out light work |
| 2 | Ambulatory and capable or all self-care but unable to carry out any work; up and about more than 50% waking hours. |
| 3 | Capable of only limited self-care; confined to bed or chair more than 50% of waking hours. |
| 4 | Completely disabled; cannot carry out any self-care; totally confined to bed or chair. |

From: Who Handbook for Reporting Results of Cancer Treatment (1979)
